# Impact of cagPAI and T4SS on the Inflammatory Response of Human Neutrophils to *Helicobacter pylori* Infection

**DOI:** 10.1371/journal.pone.0064623

**Published:** 2013-06-03

**Authors:** Norma Angélica Sánchez-Zauco, Javier Torres, Gloria Erandi Pérez-Figueroa, Lourdes Álvarez-Arellano, Margarita Camorlinga-Ponce, Alejandro Gómez, Silvia Giono-Cerezo, Carmen Maldonado-Bernal

**Affiliations:** 1 Unidad de Investigación en Enfermedades Oncológicas, Hospital Infantil de México Federico Gómez, SS. Mexico City, México; 2 Unidad de Investigación en Enfermedades Infecciosas, Centro Médico Nacional Siglo XXI, IMSS. Mexico City, México; 3 Laboratorio de Neuroinmunología, Departamento de Medicina Molecular y Bioprocesos, Instituto de Biotecnología, UNAM. Cuernavaca, Morelos, México; 4 Laboratorio de Bacteriología Médica, Escuela Nacional de Ciencias Biológicas-IPN, Mexico City, México; Veterans Affairs Medical Center (111D), United States of America

## Abstract

*Helicobacter pylori* contains a pathogenicity island, cagPAI, with genes homologous to components of the type IV secretion system (T4SS) of *Agrobacterium tumefaciens*. The T4SS components assemble a structure that transfers CagA protein and peptidoglycan into host epithelial cells, causing the increased release of interleukin 8 (IL8) from the cells. The Toll-like receptors on neutrophils recognize *H. pylori,* initiating signaling pathways that enhance the activation of NF-κB. However, the roles of cagPAI and T4SS in the inflammatory response of neutrophils are unknown. We evaluated the participation of cagPAI and T4SS in the response of human neutrophils to *H. pylori* infection. Neutrophils were isolated from the blood of healthy donors and infected with *H. pylori* cagPAI^+^, cagPAI^–^, and cagPAI mutant strains *virB4* ^–^ and *virD4* ^–^. Whereas cagPAI^+^ strain 26695 induced the greatest IL8 production, a proinflammatory response, cagPAI^–^ strain 8822 induced the greatest IL10 production, an anti-inflammatory response. In contrast, the *virB4* ^–^ and *virD4* ^–^ mutant strains produced significantly more of the two proinflammatory cytokines IL1β and tumor necrosis factor αthan the cagPAI^+^ strain 26695. We observed that *H. pylori* downregulated the expression of TLRs 2 and 5 but upregulated TLR9 expression in a cagPAI and T4SS-independent manner. These results show for the first time that the response of human neutrophils to *H. pylori* may vary from a pro-inflammatory to an anti-inflammatory response, depending on cagPAI and the integrity of T4SS.

## Introduction

Infection of the gastric mucosa by *Helicobacter pylori* is one of the most common bacterial infections worldwide. *H. pylori* infection always causes inflammation in the gastric mucosa, followed by humoral and cellular immune responses, and is associated with the development of peptic ulcer, gastric cancer, and lymphoma of mucosa-associated lymphoid tissue (MALT lymphoma) [1]. The inflammatory response induced by *H. pylori* is the key event in its pathogenesis, and the innate immune response in the gastric mucosa is triggered by several components, including host cell pattern recognition receptors. Pattern recognition receptors are expressed on epithelial cells and on neutrophils, and include the Toll-like receptors (TLRs) and Nod-like receptors [2], and the ligands for most receptors of the TLR family have already been identified [3]. We recently demonstrated that in human neutrophils, *H. pylori* can induce interleukin 8 (IL8) and IL10 production by the activation of TLR2 and TLR4 [4].

Neutrophils play a major role in the defense of mucosal surfaces against infections and in the pathogenesis of several inflammatory diseases. When neutrophils are called to fight infections in the lumen of the respiratory or the gastrointestinal tract, they leave the blood stream, traverse the endothelium, to finally reach the luminal side of the epithelium [5]. Although the mechanisms of response of neutrophils to *H. pylori* are not completely elucidated, its importance in mucosal damage and disease has been well documented. It is known that *Helicobacter* infection induces a prominent neutrophilic infiltration to the gastric mucosa, and it has been observed that the extent of mucosal injury is related to the degree of this neutrophil infiltration [6], [7]. It has been shown that depletion of neutrophils in Helicobacter-infected IL-10−/− mice decreased the severity of gastritis, modulated the Helicobacter-specific Th1 immune response, and delayed the clearance of bacteria from the stomach [8]. Also, it has been suggested that reactive oxygen intermediates, a product of neutrophil activation, play an important role in the pathogenesis of the disease [9].

*H. pylori* is a highly diverse species, with a high rate of DNA recombination, and the emergence of recombinant strains, the deletion of virulence genes, and point mutations may partially account for the complex variety of phenotypic responses in infected patients [10]–[12]. The cagPAI is a cluster of genes of approximately 40 kb, with seven of these genes, *virB4, virB7, virB8, virB9, virB10, virB11*, and *virD4*, homologous to the gene components of the type IV secretion system (T4SS) of *Agrobacterium tumefaciens* [10], [13], [14]. The T4SS components assemble as a syringe-like structure specialized for the transfer of bacterial components, such as CagA protein and peptidoglycan, into host epithelial cells [15], [16]. The cagPAI-positive strains are implicated in the increased release of various inflammatory cytokines from the host cells, including IL8 [10], [17], whereas an isogenic mutant with a defect in T4SS is unable to elicit this response [18]. Several genes of cag-PAI seem to be involved in this inflammatory response, by activating transcription factors such as activation protein-1 (AP-1) and NF-κB, leading to the increased expression of cytokines and chemokines [10], [19], [20]. Some reports have suggested that cagPAI is not the only *H. pylori* factor that promotes IL8 secretion [21], and even more significantly, cagPAI-negative strains may also induce IL8 production by epithelial cells, such as the MKN45, AGS, and KATO III cell lines [22], [23]. In fact, mucosal IL8 levels in patients infected with cagPAI-negative strains may be higher than the mucosal levels in patients infected with cagPAI-positive strains [17]. These observations suggest that *H. pylori* virulence factors other than cagPAI are involved in IL8 production and in the increased mucosal inflammatory response evoked. In this context, *in vitro* experiments have indicated that IL8 is produced after the attachment of *H. pylori* to the epithelial cells, regardless of the presence of cagPAI [21], [24]. It has been suggested that some outer membrane proteins are additional virulence factors, able to elicit a mucosal inflammatory response [24], [25].

In this work, we evaluated the role of *H. pylori* cagPAI-positive and mutant strains defective in T4SS (*virB4* and *virD4* knockout strains) in the inflammatory response of human neutrophils. To this end, we tested the ability of *H. pylori* strains with either intact or modified cagPAI to induce cytokine production in human neutrophils and their ability to affect the expression of TLRs.

## Materials and Methods

### Ethics Statement

This research protocol was approved by the Research and Ethics Committee of the Hospital de Pediatría del Centro Médico Nacional sXXI del Instituto Mexicano del Seguro Social (Registration R-2005-3603-50). The peripheral blood of healthy donors was obtained from the Blood Bank at the same Institution. The bags of blood used in the study were destined by the bank of blood for waste by excess of filling for this reason the letter of consent was not necessary.

### Neutrophil Isolation

Neutrophils were obtained from healthy donors seronegative for *H. pylori* when screened with the *H. pylori* Rapid Test (Diagnomex S.A. DE C.V.) and confirmed with a previously validated enzyme-linked immunosorbent assay (ELISA) [26].

Neutrophils were isolated with density-gradient LymphoPrep™ (Nycomed, Oslo, Norway) and centrifuged at 800×g for 30 min at room temperature. After lysis of the erythrocytes, the neutrophils were washed and resuspended on RPMI-1640 medium (Life Technologies, Gaithersburg, MD) supplemented with 2% fetal bovine serum (Gibco BRL) and penicillin–streptomycin (100 UI–100 mg/mL), and cultured at 37°C. Cell viability was tested with Trypan blue exclusion.

### Bacterial Culture and Neutrophil Infection

The *H. pylori* strains used in this study are described in Table 1. We used the *H. pylori* strain 26695 and the mutated strains knockout in *virD4* ^–^ and *virB4* ^–^ made in strain G27 as previously described [27] (kindly supplied by Nina Salama, FHCRC, Seatle, WA) and clinically isolated cagPAI^+^ and cagPAI^–^ bacteria. The bacteria were grown on Brucella agar containing 5% sheep blood and incubated at 37°C under microaerophilic conditions for 24 h. Their growth was stopped, the bacteria washed with sterile saline, and the suspension adjusted to 10^9^/mL. The neutrophils were infected at a multiplicity of infection of 100 (100 bacteria per neutrophil) and incubated in RPMI-1640 medium for 0.5, 1, 3, 6, and 24 h at 37°C. As control we included cells that were mock infected by adding only RPMI media. After infection, the cells were centrifuged and the supernatants separated and stored at –70°C until analysis. The neutrophils were analyzed for their TLR expression as described below.

**Table 1:** Genotype characteristics of *H. pylori* strains and their origins.

Wild-type strains		Genotypes	Sources
26695		cagPAI^+^, Oip A on	Donated by Dr. Nina R. Salama, Human Biology, Fred Hutchinson Cancer Research Center, Seattle, Washington.
8822		cagPAI^–^, Oip A on	Donated by Dr. Germán Aguilar Gutiérrez, National Institutes of Public Health, Cuernavaca, Morelos.
*VirB4 mutant		cagPAI^+^, Oip A on, *virB4^–^*	Donated by Dr. Nina R. Salama, Human Biology, Fred Hutchinson Cancer Research Center, Seattle, Washington.
*VirD4 mutant		cagPAI^+^, Oip A on, *virD4^–^*	Donated by Dr. Nina R. Salama, Human Biology, Fred Hutchinson Cancer Research Center, Seattle, Washington.
Clinical isolates			
256	cagPAI^+^, Oip A off		Duodenal ulcer
261	cagPAI^+^, Oip A on		Gastric ulcer
370	cagPAI^–^, Oip A on		Recurrent abdominal pain

* Mutants were made in G27 strain (Pinto-Santini DM, Salama NR (2009) Cag3 Is a Novel Essential Component of the *Helicobacter pylori* Cag Type IV Secretion System Outer Membrane Subcomplex. J Bacteriol 191∶7343–7352).

### Expression of TLRs by Neutrophils Infected with H. Pylori Strains

After infection with the different *H. pylori* strains, the neutrophils were resuspended at 5×10^5^ cells/mL in blocking buffer (phosphate-buffered saline containing 2% FBS, 2% rabbit serum, 5 mM EDTA, and 0.1% sodium azide), incubated on ice for 1 h, and incubated with phyocerythrin (PE)-conjugated monoclonal antibodies CD16b–PE, CD3–PE, CD14–PE, and CD19–PE (BD Pharmingen, San Jose, CA). Another set of neutrophils was incubated with PE-conjugated anti-human TLR2 and TLR4 (Santa Cruz Biotechnology, Santa Cruz, CA) and fluorescein-isothiocyanate-conjugated anti-human TLR5 (Santa Cruz Biotechnology) and anti-human TLR9 (Imgenex Corp., San Diego, CA). The isotype controls used to exclude nonspecific staining were PE-conjugated anti-IgG2a and fluorescein-isothiocyanate-conjugated anti-IgG1 antibodies (Santa Cruz Biotechnology). The cells were incubated for 15 min in the dark, washed twice with washing buffer (phosphate-buffered saline containing 2% FBS, 5 mM EDTA, and 0.1% sodium azide), and fixed with 4% paraformaldehyde for 30 min. The cells were analyzed by flow cytometry (FACSAria, Becton-Dickinson, San Jose, CA), with 20,000 events per sample, and analyzed with the Summit software (Dako). The results are reported as fluorescence intensities and as percentages of positive cells, and the data are the medians ± intervals of percentiles of three independent experiments.

### Estimation of Cytokines Released by Neutrophils

The release of cytokines IL8, IL1β, tumor necrosis factor α (TNFα), and IL10 by neutrophils was measured with a commercial ELISA (BD Pharmingen, San Diego, CA), according to the manufacturer’s instructions. The capture antibodies were bound to 96-well microtiter plates (Greiner, Solingen, Germany) overnight at 4°C, and after they were blocked for 1 h with PBS–10% fetal calf serum, the test samples were added and incubated for 2 h. Biotinylated anti-IL8, anti-IL1β, anti-TNFα, and anti-IL10 were added and incubated for 1 h, and the reactions were visualized with an avidin–peroxidase substrate buffer. The sensitivity of the assays was 3.1 pg/mL for IL8, 3.9 pg/mL for IL1β, and 7.8 pg/mL for TNFα and 3.2 pg/mL for IL10. We used RPMI-1640 medium alone as the negative control. In all tests, a standard cytokine preparation (recombinant cytokines at defined concentrations) was used to estimate the cytokine concentrations in the samples. The data are expressed as media ± SEM of at least five independent experiments.

### Statistical Analysis

Differences in the kinetics of induction of cytokines among all *H. pylori* tested strains were analyzed using two-way analysis of variance (ANOVA), independent variables included were strains and times of infection. Differences between each of the strains of infection were analyzed with the Student–Newman–Keuls post-hoc test. The expression of TLRs after infection with the strains was tested by flow-cytometric and results expressed as medians and percentiles, and differences between strains analyzed using the Kruskal–Wallis one-way test. Differences were considered statistically significant at *p*<0.05.

## Results

**Figure 1:**
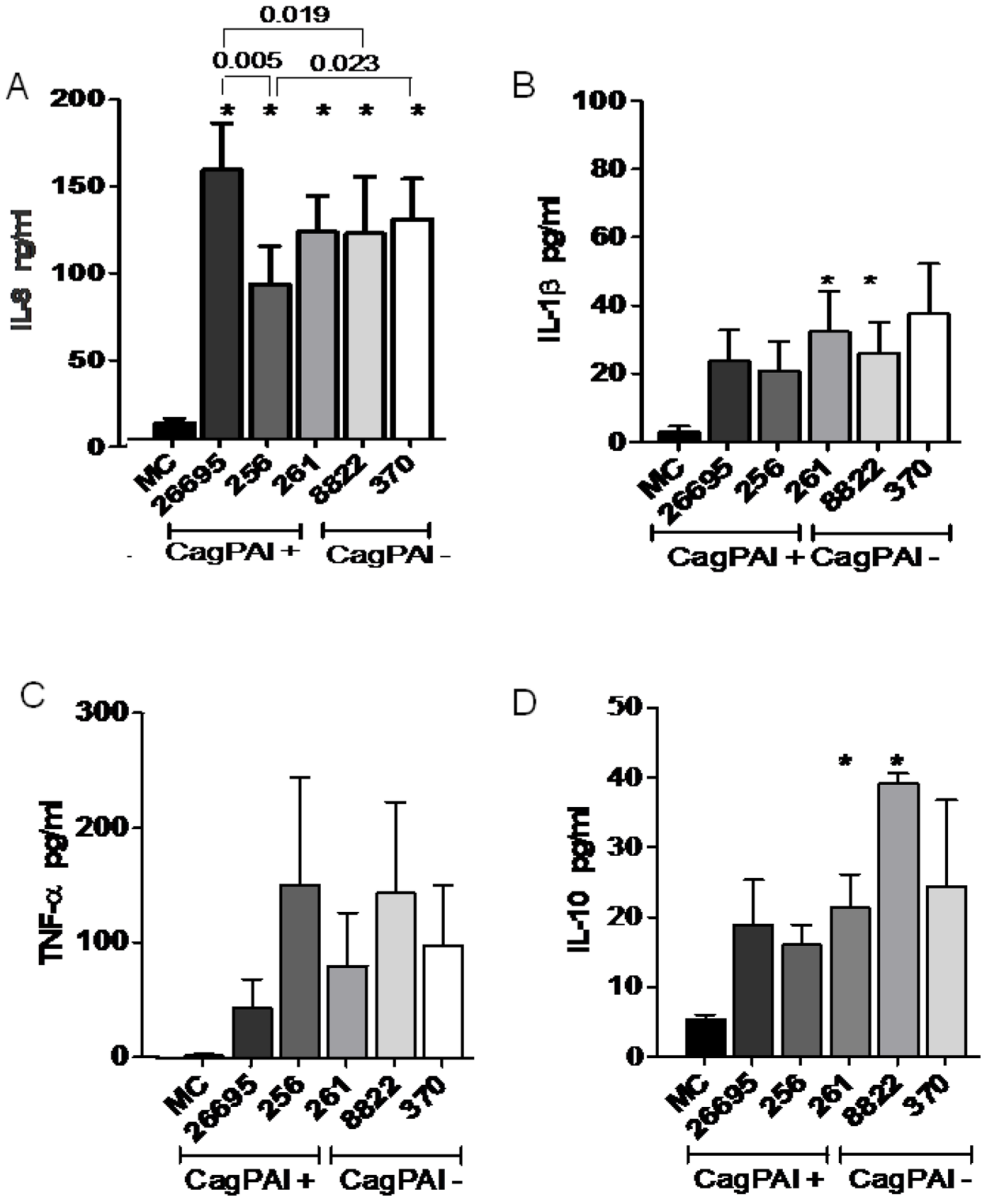
*Helicobacter pylori* induced an increase in cytokine expression in human neutrophils, regardless of the status of its cagPAI. A) Culture supernatants 24 h after infection with cagPAI^+^ or cagPAI^–^ *H. pylori* strains were analyzed for IL8 secretion with an ELISA. cagPAI^+^ strain 26695 induced more IL8 than cagPAI^–^ strain 8822, and the lack of functional OipA protein in the cagPAI^+^256 strain was associated with a reduction in the amount of IL8 induced. B) Clinically isolated cagPAI^+^ strain 261 and cagPAI^–^ strain 8822 induced the highest levels of IL1β. C) The expression of TNFα did not differ in the cells infected with these two strains. D) cagPAI^+^ strain 261, isolated from a gastric ulcer, and cagPAI^–^ strain 8822 induced the greatest amounts of IL10. The data shown are from five independent experiments performed in duplicate. The data from these five experiments were pooled and analyzed by ANOVA to assess their statistical significance. *P<0.05 for *H. pylori*-infected neutrophils versus uninfected neutrophils. Differences between the strains were analyzed with the Student–Newman–Keuls test and were considered statistically significant when *P<0.05. MC = mock control.

*Helicobacter pylori* induced the production of cytokines by neutrophils as early as 0.5 h after infection, although only after 3 h did the concentration began to reach significance, and the differences between strains were significant 24 h after infection (Figures 1 and S1).

The induction of IL8 began 0.5 h after infection, and differences between the strains were already significant after 6 h, with the highest concentrations reached after 24 h (Figures 1A and S1A). The differences in IL8 expression among strains after 24 h are described in Figure 1A, which shows that all *H. pylori* strains expressed significantly higher IL8 than the mock control. The highest IL8 production was induced by cagPAI^+^ strain 26695, and the lowest by cagPAI^+^ strain 256 (Figure 1A). The control cagPAI^+^ strain 26695 induced significantly higher IL8 than the control cagPAI^–^ strain 8822 (p<0.05). When the strains were tested in AGS cells, the cagPAI^+^ strain elicited significantly higher expression of IL8 than the cagPAI^–^ strain at all times tested (Figure S2).

Only clinical isolates cagPAI^+^ strain 261 and cagPAI^–^ strain 8822 induced significant release of IL1β compared with the mock control (p<0.05; Figure 1B). Although all the tested *H. pylori* strains induced the release of more TNFα than was released by the mock control, the differences were not significant (Figure 1C). In contrast, only strains 261 and 8822 induced a significant release of IL10 (Figure 1D).

### Integrity of H. Pylori T4SS Influences the Cytokine Response of Human Neutrophils

**Figure 2:**
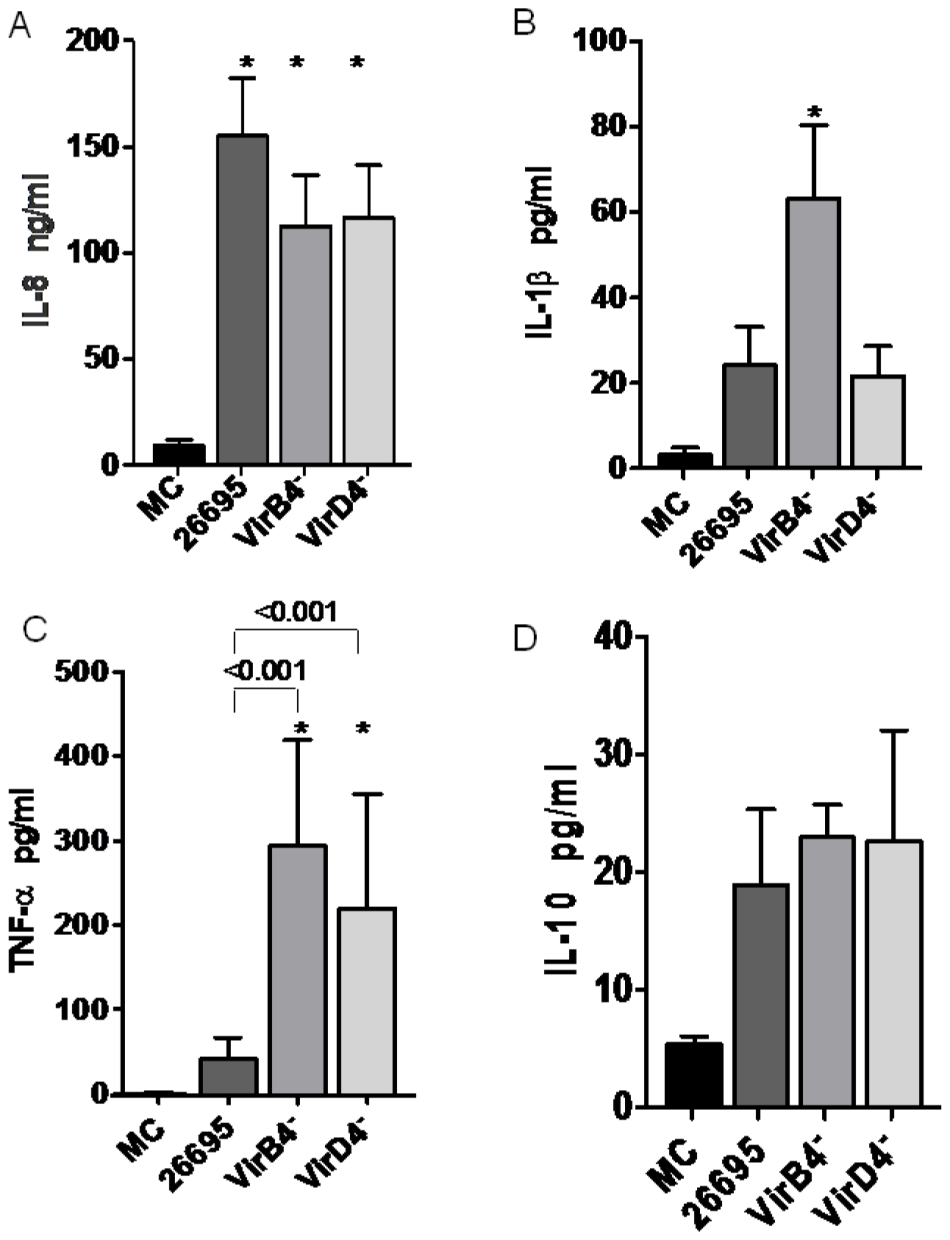
Effect of *H. pylori* T4SS integrity on cytokine induction in human neutrophils. A) Strains lacking the genes that encode virB4 or virD4 protein in the cagPAI did not modify neutrophil IL8 production compared with the cagPAI^+^26695 reference strain. B) The *virB4^–^* strain induced high levels of IL1β compared with the other mutant *virD4^–^* strain or the 26695 cagPAI^+^ strain. C) High TNFα expression was induced in neutrophils by both mutant strains compared with the cagPAI^+^ strain 26695. D) The *virB4^–^* and *virD4^–^* strains induced the expression of similar amounts of IL10, but these did not differ significantly from that induced by cagPAI^+^ strain 26695. The data shown are from five independent experiments performed in duplicate. The data from these five experiments were pooled and assessed with ANOVA to determine their statistical significance. *P<0.05 for *H. pylori*-infected neutrophils versus uninfected neutrophils. Differences between the strains were analyzed with the Student–Newman–Keuls test, and differences were considered statistically significant when *P<0.05. MC = mock control.

A functional bacterial T4SS is necessary for the induction of IL8 in gastric epithelial cells, but we do not know whether this is also true of neutrophils. To address this issue, we infected neutrophils with the *H. pylori* strain 26695 and the mutant strains in *virB4^–^*, in which the assembly of T4SS is inactivated, or *virD4^–^*, which expresses a defective T4SS. We found that both mutant strains were able to induce IL8 secretion at a level similar to that induced by strain 26695 (Figures 2A and S3A), suggesting that a functional bacterial T4SS is not required for the induction of IL8 in neutrophils.

However, whereas strain 26695 and mutant strain *virD4* ^–^ were unable to increase the release of IL1β significantly, the mutant strain *virB4^–^* induced significantly higher levels of this interleukin (Figures 2B and S3B). When the production of TNFα was tested, both the *virB4* ^–^ and *virD4* ^–^ mutants induced greater secretion of TNFα than the 26695 strain (Figures 2C and S3C). Neither 26695 nor the *virB4* ^–^ and *virD4* ^–^ mutants significantly induced the release of IL10 by neutrophils (Figures 2D and S3D).

### Helicobacter Pylori Infection Modifies the Expression of TLRs Regardless of the Status of cagPAI and T4SS

**Figure 3:**
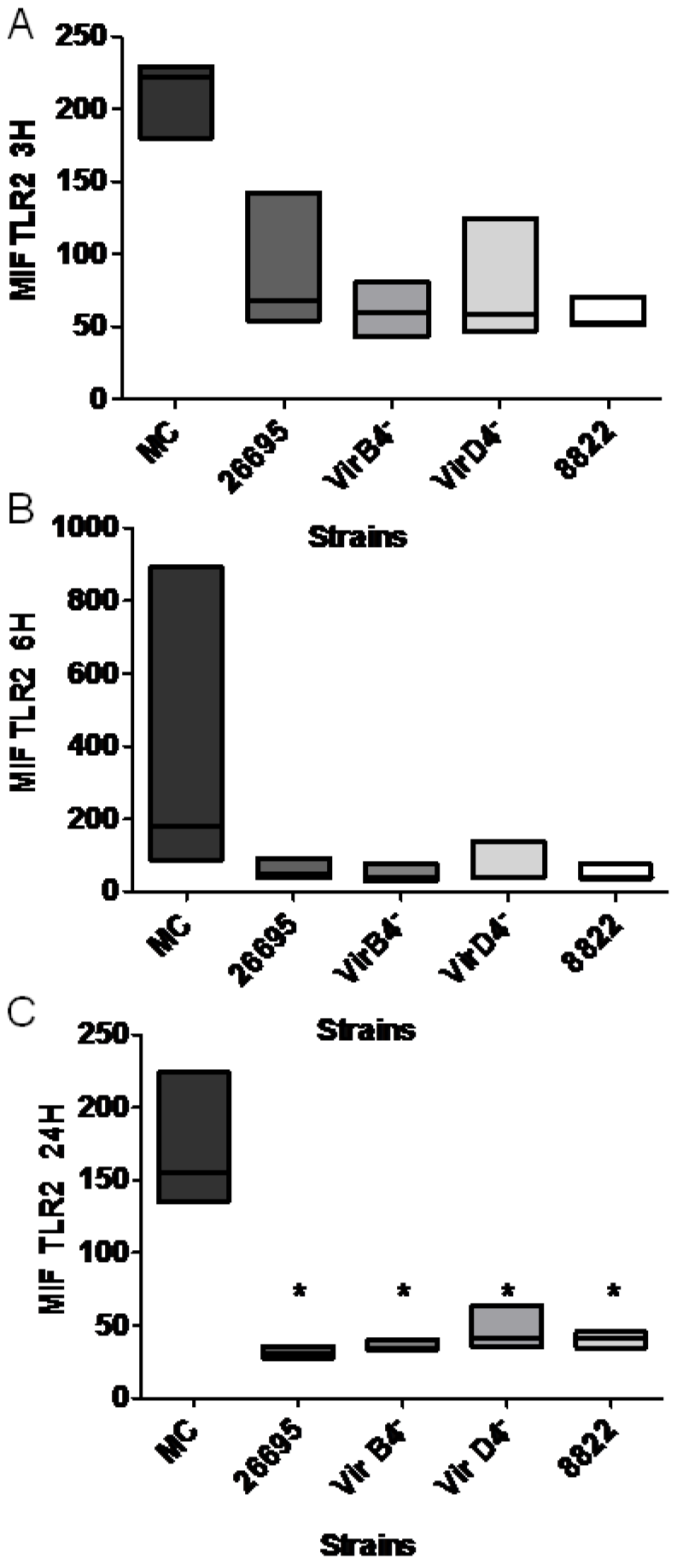
*Helicobacter pylori* cagPAI^+^ strain, T4SS mutants, and a cagPAI^–^ strain downregulated neutrophil TLR2 expression. Mean fluorescence intensity (MFI) of TLR2 on neutrophils 3, 6, and 24 h after infection (A, B, and C, respectively) with cagPAI^+^ strain 26695, T4SS mutants (*virB4^–^* and *virD4^–^*), or cagPAI^–^ strain 8822. The data shown are from three independent experiments and line represent median. Flow cytometry results were obtained as medians ± intervals of percentiles and analyzed with the Kruskal–Wallis one-way test. Differences were considered statistically significant when *P<0.05. MC = mock control.

**Figure 4:**
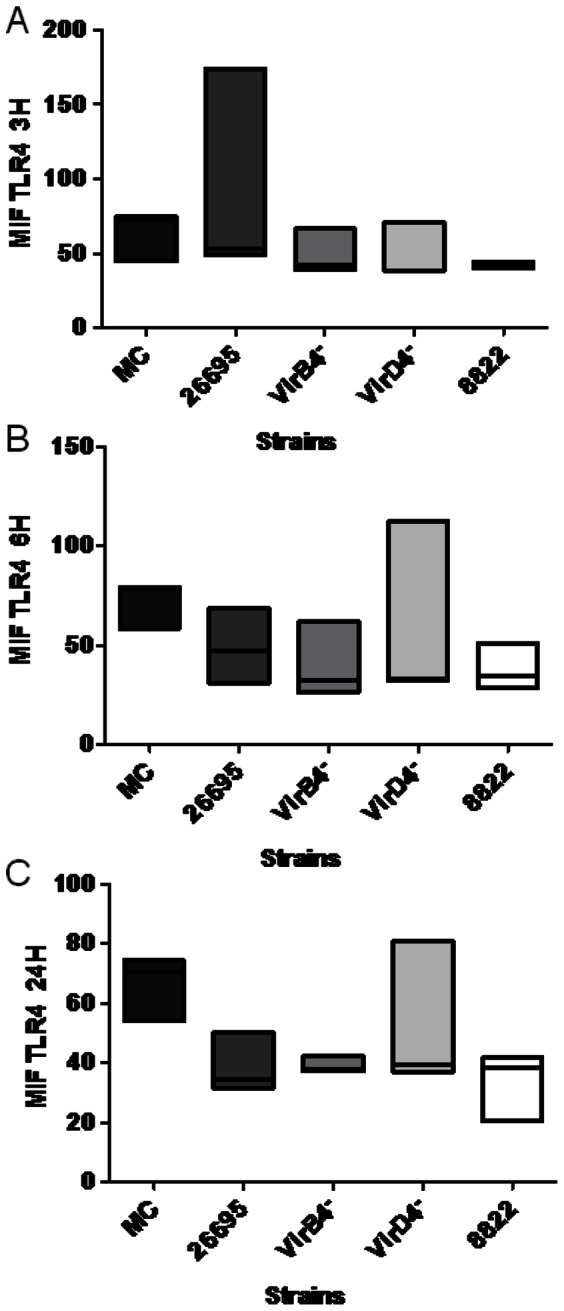
*Helicobacter pylori* cagPAI^+^ strains and a cagPAI^–^ strain did not induce changes in TLR4 expression. Mean fluorescence intensity (MIF) of TLR4 on neutrophils 3, 6, and 24 h after infection (A, B, and C, respectively) with cagPAI^+^ strain 26695, T4SS mutants (*virB4^–^* and *virD4^–^*), or cagPAI^–^ strain 8822. The data shown are from three independent experiments and line represent median. Flow cytometry results were obtained as medians ± intervals of percentiles and analyzed with the Kruskal–Wallis one-way test. Differences were considered statistically significant when*P<0.05. MC = mock control.

Human neutrophils express TLR1–TLR9, except TLR3, and some of these participate in the inflammatory response to *H. pylori* infection [4]. We investigated whether the expression of these receptors was modified by infection with *H. pylori* strains that carried an intact or defective T4SS or lacked cagPAI. In a kinetic study, we found that the strain 26695 and the *virB4^–^* and *virD4^–^* mutants reduced TLR2 expression 3, 6, and 24 h after infection (Figure 3A, 3B, and 3C respectively), although this reduction was only significant after 24 h for all three strains. Infection with cagPAI^–^ strain 8822 caused a similar reduction in TLR2 expression, suggesting that neither T4SS nor cagPAI is necessary for this *H. pylori* activity. We also studied the expression of TLR4 (Figure 4A, 4B, and 4C) and found that the behavior of the four tested strains (26695, *virB4^–^*, *virD4^–^*, and 8822) was similar, causing no significant reduction in TLR4 expression, even 24 h after infection.

**Figure 5:**
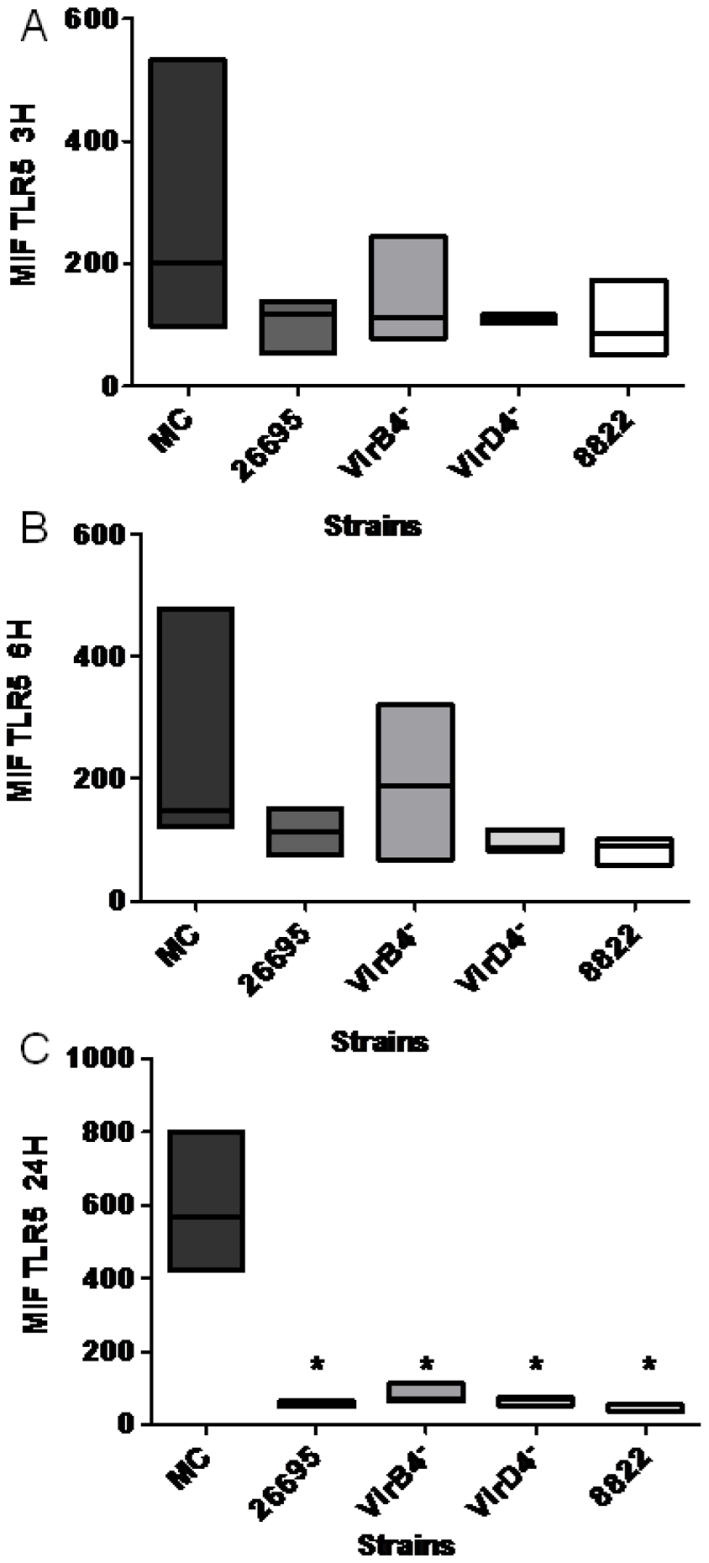
*Helicobacter pylori* cagPAI^+^ strain, T4SS mutants, and a cagPAI^–^ strain downregulated neutrophil TLR5 expression. Mean fluorescence intensity (MFI) of TLR5 on neutrophils 3, 6, and 24 h after infection (A, B, and C, respectively) with cagPAI^+^ strain 26695, T4SS mutants (*virB4^–^* and *virD4^–^*), or cagPAI^–^ strain 8822. The data shown are from three independent experiments and line represent median. The results were obtained as medians ± intervals of percentiles and analyzed using the Kruskal–Wallis one-way test. Differences were considered statistically significant when *P<0.05. MC = mock control.

**Figure 6:**
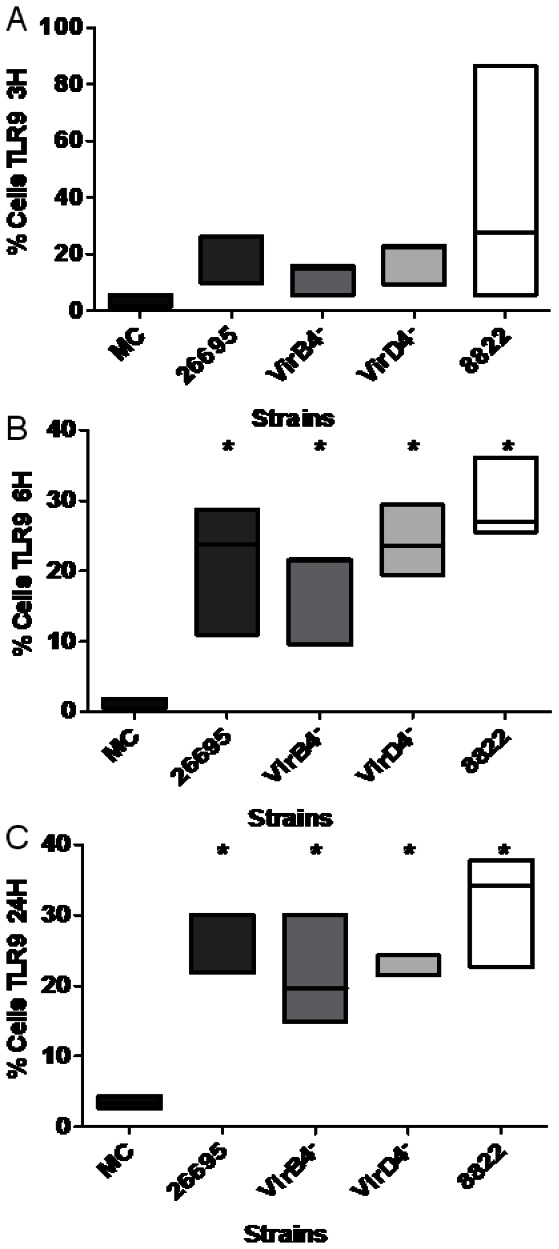
*Helicobacter pylori* cagPAI^+^ strain, T4SS mutants, and a cagPAI^–^ strain upregulated TLR9 expression. The percentage of neutrophils expressing TLR9 3, 6, and 24 h after infection (A, B, and C, respectively) with cagPAI^+^ strain 26695, T4SS mutants (*virB4^–^* and *virD4^–^*), and cagPAI^–^ strain 8822. The data shown are from three independent experiments and line represent median. The results were obtained as medians ± intervals of percentiles and analyzed with the Kruskal–Wallis one-way test. Differences were considered statistically significant when *P<0.05. MC = mock control.

The results for TLR5 showed a different pattern, insofar as its expression 3 and 6 h after infection with all four strains was similar to that observed in mock control (Figure 5A and 5B). However, after 24 h, cells not infected with *H. pylori* showed an increase in TLR5 expression, whereas the cells infected with any one of the four strains showed significantly reduced TLR5 expression (Figure 5C). In the absence of *H. pylori* infection, TLR9 was expressed negligibly, even after 24 h (Figure 6). In contrast, infection with strain 26695, *virB4^–^*, *virD4* ^–^, or 8822 resulted in a significant increase in the expression of TLR9 6 h and 24 h after infection (Figure 6B and 6C).

## Discussion

*Helicobacter pylori* is a bacterium that colonizes the gastric mucosa, causing innate and adaptive immune responses in the host. The initial inflammatory response is characterized by the infiltration of polymorphonuclear cells, which are the key cells in the elimination of microorganisms [28].

Neutrophils are a central component of the innate immune response, acting as primary responders by rapidly migrating to the infected tissues and subsequently using potent effector mechanisms, such as phagocytosis, the production of reactive oxygen species, and the release of inflammatory mediators and antimicrobial substances. Neutrophils use TLRs to detect the presence of bacteria, which recognize the molecular patterns on the bacterial surfaces, allowing an effective antimicrobial response [4].

IL8 is a very important proinflammatory chemokine and is involved in the chemotaxis of neutrophils, which elicits a quick response to the infectious agents. IL8 is also an important factor in the immunopathogenesis of peptic ulcer and gastric carcinogenesis [29]. It has been suggested that the expression of IL8 is induced more strongly by more virulent *H. pylori* genotypes, such as the cagPAI^+^ strains [30], [31]. However, in our study, we observed that in human neutrophils, *H. pylori* cagPAI^+^ and cagPAI^–^ strains induced IL8 to similar extents, which suggests that the response to *H. pylori* by neutrophils differs from the response of epithelial cells, in which cagPAI^+^ strains usually induce stronger IL8 expression [23]. Although we saw no significant differences in the induction of IL8 between the cagPAI^+^ and cagPAI^–^ strains, among the cagPAI^+^ strains, those that induced IL8 most strongly (strains 26695 and 261) had the *oipA* gene on, in contrast to the strain causing the lowest IL8 induction (strain 256), in which *oipA* was off (unpublished data). This suggests that OipA participates in the induction of IL8 in neutrophils, similar to its action reported in epithelial cells [18]. In contrast, the induction of IL8 in neutrophils infected with the mutant strain *virB4^–^* or *virD4^–^* was lower than that observed in cells infected with the strain 26695, although the differences were not significant. It is known that partial deletions within the cagPAI result in a defective T4SS, with an impact on bacterial virulence and on the clinical outcome [19]. Furthermore, the *virB4* gene is homologous to the *dupA* gene, which has also been associated with the induction of IL8 in *in vitro* tests [28], [29]. Other *in vitro* studies have also shown that CagE (HP0544) is homologous to the ATPase VirB4, which is essential for the induction of IL8 [10]. These data suggest that VirB4 and VirD4 may have a cagPAI-independent capacity to induce IL8.

It has been reported that the cagPAI^+^ strains induce the release of IL1β in the gastric mucosa [32]. However, we observed that in neutrophils, the cagPAI^+^ and cagPAI^–^ strains induced similar amounts of IL1β. Furthermore, the mutant *virB4^–^* elicited significantly greater production of IL1β than strain 26695, suggesting that the induction of IL1β in neutrophils is an activity independent of the integrity of T4SS. We speculate that the *vir*B4^–^strain, in which the assembly of T4SS is inactivated, is forming a defective T4SS resulting in an altered presentation of T4SS components or even of other surface molecules like LPS to TLRs, hence causing an altered induction of inflammatory mediators. In agreement with this, a recent study reported that epithelial cells challenged with heat killed *H. pylori* (no activity of the T4SS) induced significantly more cytokines than live cagPAI strains (exhibiting a functional T4SS), and authors also suggested this might be due to change in presentation of surface antigens like LPS to epithelial cells [33]. IL1β has been described as an autocrine survival factor or a paracrine product of neutrophils stimulated with lipopolysaccharide (LPS), producing a more effective inflammatory response [34]. It has also been reported that there is no recruitment of neutrophils in IL1β- and IL1 receptor (IL1R)-deficient mice [35]. This suggests that *virB4*-deficient strains could cause stronger recruitment of neutrophils to the infection site.

TNFα is a cytokine that participates in the inflammatory response to *H. pylori* and plays an important role in tumorigenesis. In this study, we found that none of the reference and clinical strains induced significant secretion of TNFα in neutrophils, which would argue that production of TNFα by neutrophils is not relevant in human *H. pylori* infection. This does not preclude the production of TNFα by other cells; in fact, *H. pylori* encodes proteins with the ability to induce TNFα in gastric cell in a cagPAI independent maner [36]. It was interesting to observe that in contrast to clinical and reference strains, the mutant strains *VirB4-* and *VirD4-* were able to induce the significant release of TNFα. The observation that *virB4-* strain expresses a phenotype more efficient to up-regulate the production of both IL1β and TNFα is hard to explain since this strain is now forming a defective T4SS; a probability would be that the mutant produces an irregular T4SS structure, which is recognized differently by the neutrophils.

Not all the *H. pylori* strains tested were able to induce IL10 in human neutrophils, as was also observed for IL1β. In fact, the same cagPAI^+^ strain (261) and cagPAI^–^ strain (8822) that induced IL1β release also induced IL10 release. The production of IL10 may be important for the persistence of *H. pylori* infection because IL10 inhibits the expression of IL8 and the activation of NF-κB, favoring *H. pylori* growth [8].

The cytokines analyzed in this study are the products of activated transcription factors such as NF-κB and AP-1, which are usually activated through signal cascades initiated by TLRs [3]. Therefore, it was relevant to explore the effects of cagPAI and T4SS of *H. pylori* on the expression of the TLRs by human neutrophils infected with *H. pylori* strains expressing intact cagPAI and strains defective in T4SS. TLRs play an important role in the immunological detection of *H. pylori* infection because they detect the different pathogen-associated molecular patterns (PAMPs) expressed by the bacterium, like LPS, flagellar proteins, or specific DNA sequences. The activation of the TLRs on neutrophils also causes the inhibition of apoptosis, suggesting a role for the TLRs in the regulation of neutrophil longevity [34].

Major studies of the inflammation induced by *H. pylori* have been performed in gastric cancer cell lines or in animal models [23], [37]. Cell lines express different levels of TLRs on their surfaces [38], [39]. Therefore, the recognition of *H. pylori* by neutrophils differs from their recognition by other cells and may produce a different inflammatory response.

We observed that *H. pylori* reduced the expression of TLR2. The *H. pylori* ligands recognized by TLR2 are LPS [40] and heat shock protein 60 [41], and our results suggest that its interaction with these ligands downregulates the expression of TLR2 in human neutrophils. In contrast, it has been reported that in gastric epithelial cells, *H. pylori* infection was associated with an increase in TLR2 expression [40]. It seems then that *H. pylori* infection affects the expression of TLR2 differently in the different cells present in the gastric mucosa.

We found that neither the cagPAI^+^ nor cagPAI^–^ *H. pylori* strains altered TLR4 expression in human neutrophils. Moreover, no differences were found in this regard between the cagPAI^+^ and mutant *virB4^–^* and *virD4^–^* strains. It has been reported that TLR4 from mice and guinea pigs, but not from humans, recognizes tetra- and penta-acylated LPS, and that the LPS from *H. pylori* is penta-acylated [42], which could partially explain why we observed no changes in the expression of TLR4 after infection with the different *H. pylori* strains. In fact, it was recently reported that TLR2 and TLR5, but not TLR4, are required for *H. pylori*-induced NF-κB activation and for the induction of chemokine expression in epithelial cells [39].

We found that in neutrophils without *H. pylori* infection, the expression of TLR5 increased significantly after 24 h culture, whereas infection with cagPAI^+^, cagPAI^–^, or the *virB4* ^–^ or *virD4* ^–^ mutant strain resulted in the significant inhibition of TLR5 expression. By inhibiting TLR5 expression, *H. pylori* would prevent the activation of phagocytosis, IL8 release, and the increased respiratory burst associated with TLR5 activation [5]. TLR5 in human gastric epithelial cell lines recognizes flagellin, which induces NF-κB activation and the expression of several chemokines, mainly IL8 and GRO-α, which attract neutrophils to the infection site [39], [43]. The infection of human neutrophils with *H. pylori* resulted in a significant increase in the expression of TLR9, and this increase was observed with all the strains tested, regardless of the presence of cagPAI. TLR9 is mainly an endosomal receptor and is usually not expressed on the cell surface [43]. However, here we present evidence suggesting that TLR9 is expressed on the surfaces of neutrophils. This is relevant because the strong expression of TLR9 may increase the sensitivity of CpG-DNA recognition, and the phagocytosis of *H. pylori*, increasing the inflammatory response during infection [44].

In summary, we have demonstrated that human neutrophils respond to *H. pylori* infection by producing IL8, regardless of the status of cagPAI and of the integrity of T4SS. In contrast, not all strains induce the significant release of IL1β, TNFα or IL10. Whereas cagPAI^+^ strain 26695 induced the strongest IL8 production, a proinflammatory response, cagPAI^–^ strain 8822 induced the strongest IL10 production, an anti-inflammatory response. Interestingly, *virB4^–^* strain induced the highest production of IL1β and TNFα, implying that this strain increases the pro-inflammatory signals of *H. pylori* on human neutrophils and we also show that all the *H. pylori* strains tested downregulated the expression of TLR2 and TLR5, and only the expression of TLR9 was upregulated, independent of the cagPAI status and of the integrity of T4SS. Of importance is the finding that the effect on the expression of TLRs was independent of the cagPAI status, we hypothesized that since neutrophils phagocyte *H. pylori*, the interaction of bacteria with its cell membrane, including via the T4SS is minimized and in the response to the infection the role of TLRs and *H. pylori* antigens other than those presented by cagPAI is more relevant than in epithelial cells. To our knowledge, this is the first work to investigate the effects of *H. pylori* cagPAI and T4SS in inflammatory response of human neutrophils.

## Supporting Information

Figure S1 ***Helicobacter pylori*** **induced increases in human neutrophil IL8, ILβ, TNFα, and IL10, regardless of the status of cagPAI.** Culture supernatants 0.5, 1, 3 and 6 h after infection with cagPAI^+^ or cagPAI^–^ *H. pylori* strains were analyzed for cytokine secretion with ELISAs. A) cagPAI^–^ strain 8822 induced the secretion of more IL8 than the other strains at 3 h, and all strains induced similar increases in IL8 at 6 h. B) From 0.5 h to 6 h, clinically isolated cagPAI^+^ strain 261 and cagPAI^–^ strain 8822 induced the highest levels of IL1β. C) The production of TNFα did not differ in the cells infected with the two strains at any time. D) The cagPAI^+^ strain 261 and cagPAI^–^ strain 8822 induced the greatest amounts of IL10 from 0.5 h to 6 h. The data shown are from five independent experiments performed in duplicate. The data from these five independent experiments were assessed with ANOVA to determine their statistical significance. *P<0.05 for *H. pylori*-infected neutrophils versus uninfected neutrophils. Differences between strains were analyzed with the Student–Newman–Keuls test and were considered statistically significant when *P<0.05. MC = mock control.
(TIF)

Figure S2 **IL8 expression induced in AGS cells by cagPAI^+^ and cagPAI^–^** ***H. pylori*** **strains.** In this study, the control used for the induction of IL8 was AGS cells infected with the two different types of strains of *H. pylori*, strain 26695 (cagPAI^+^) and strain 8822 (cagPAI^–^), in a kinetic assay with a maximum period of infection of 24 h. This corroborated the finding that the cagPAI^+^ strain induced considerably higher production of IL8 than the cagPAI^–^ strain, and that this IL8 production was dependent on the time of infection. The data shown are from three independent experiments performed in duplicate. The data were assessed with ANOVA to determine their statistical significance. *P<0.05 for *H. pylori*-infected AGS cells versus uninfected cells. Differences between strains were analyzed with the Student–Newman–Keuls test and were considered statistically significant when *P<0.05. MC = mock control.
(TIF)

Figure S3 **Effect of the integrity of** ***H. pylori*** **T4SS on cytokine production in human neutrophils.** A) Strains lacking the cagPAI genes that encode VirB4 or VirD4 protein induced neutrophil IL8 secretion 6 h after infection, which did not differ from the secretion of IL8 induced by the cagPAI^+^ strain 26695. B) At 3 h and 6 h after infection, the *virB4^–^* strain induced a higher level of IL1β secretion than the other mutant *virD4^–^* strain or the wild-type cagPAI^+^ strain. C) At 3 and 6 h, higher levels of TNFα secretion were induced by both mutant strains than by the cagPAI^+^ strain 26695. D) Both the *virB4^–^* and *virD4^–^* strains induced similar levels of IL10 secretion, but these did not differ significantly from that induced by cagPAI^+^ strain 26695. The data shown are from five independent experiments performed in duplicate. The data were assessed with ANOVA to determine their statistical significance. *P<0.05 for *H. pylori*-infected neutrophils versus uninfected neutrophils. Differences between strains were analyzed with the Student–Newman–Keuls test and were considered statistically significant when *P<0.05. MC = mock control.
(TIF)
