# Supplementary figures and images for: Impact of cagPAI and T4SS on the Inflammatory Response of Human Neutrophils to Helicobacter pylori Infection
Source: PLoS One. 2013 Jun 3;8(6):e64623. doi: 10.1371/journal.pone.0064623 (PMC3670914; doi:10.1371/journal.pone.0064623)

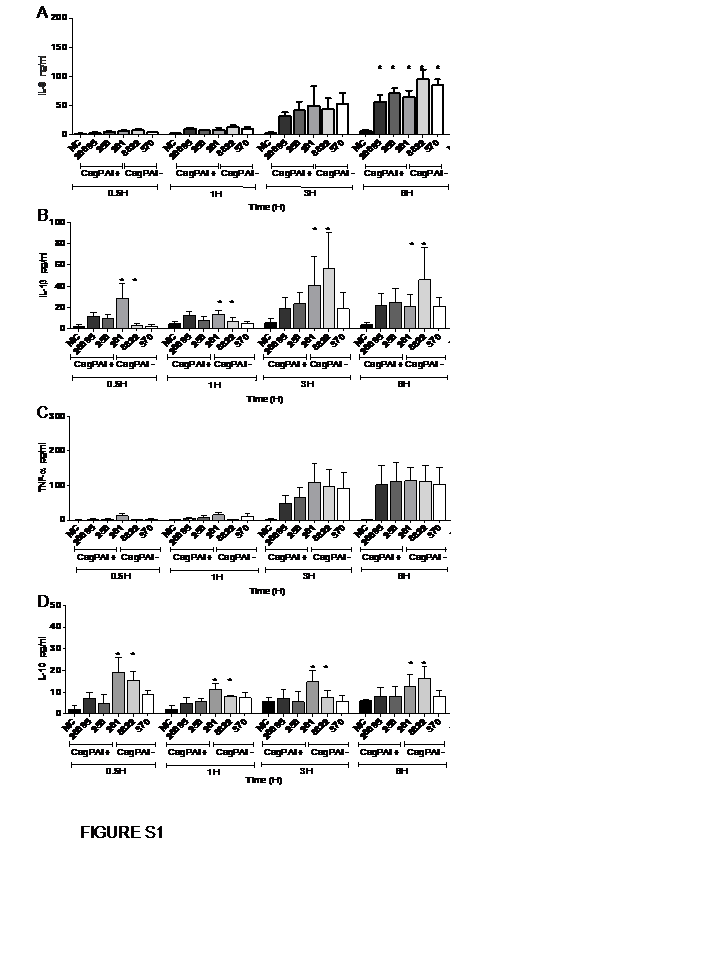

Supplement: Figure S1 — Helicobacter pylori induced increases in human neutrophil IL8, ILβ, TNFα, and IL10, regardless of the status of cagPAI. Culture supernatants 0.5, 1, 3 and 6 h after infection with cagPAI+ or cagPAI– H. pylori strains were analyzed for cytokine secretion with ELISAs. A) cagPAI– strain 8822 induced the secretion of more IL8 than the other strains at 3 h, and all strains induced similar increases in IL8 at 6 h. B) From 0.5 h to 6 h, clinically isolated cagPAI+ strain 261 and cagPAI– strain 8822 induced the highest levels of IL1β. C) The production of TNFα did not differ in the cells infected with the two strains at any time. D) The cagPAI+ strain 261 and cagPAI– strain 8822 induced the greatest amounts of IL10 from 0.5 h to 6 h. The data shown are from five independent experiments performed in duplicate. The data from these five independent experiments were assessed with ANOVA to determine their statistical significance. *P<0.05 for H. pylori-infected neutrophils versus uninfected neutrophils. Differences between strains were analyzed with the Student–Newman–Keuls test and were considered statistically significant when *P<0.05. MC = mock control. (TIF) [file pone.0064623.s001.tif]

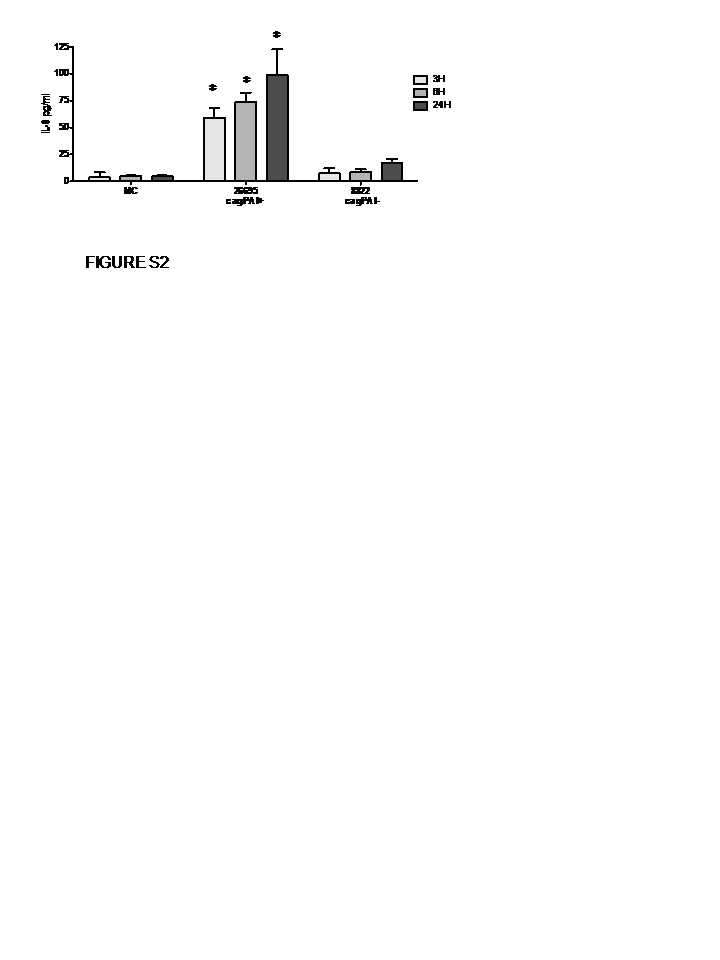

Supplement: Figure S2 — IL8 expression induced in AGS cells by cagPAI+ and cagPAI– H. pylori strains. In this study, the control used for the induction of IL8 was AGS cells infected with the two different types of strains of H. pylori, strain 26695 (cagPAI+) and strain 8822 (cagPAI–), in a kinetic assay with a maximum period of infection of 24 h. This corroborated the finding that the cagPAI+ strain induced considerably higher production of IL8 than the cagPAI– strain, and that this IL8 production was dependent on the time of infection. The data shown are from three independent experiments performed in duplicate. The data were assessed with ANOVA to determine their statistical significance. *P<0.05 for H. pylori-infected AGS cells versus uninfected cells. Differences between strains were analyzed with the Student–Newman–Keuls test and were considered statistically significant when *P<0.05. MC = mock control. (TIF) [file pone.0064623.s002.tif]

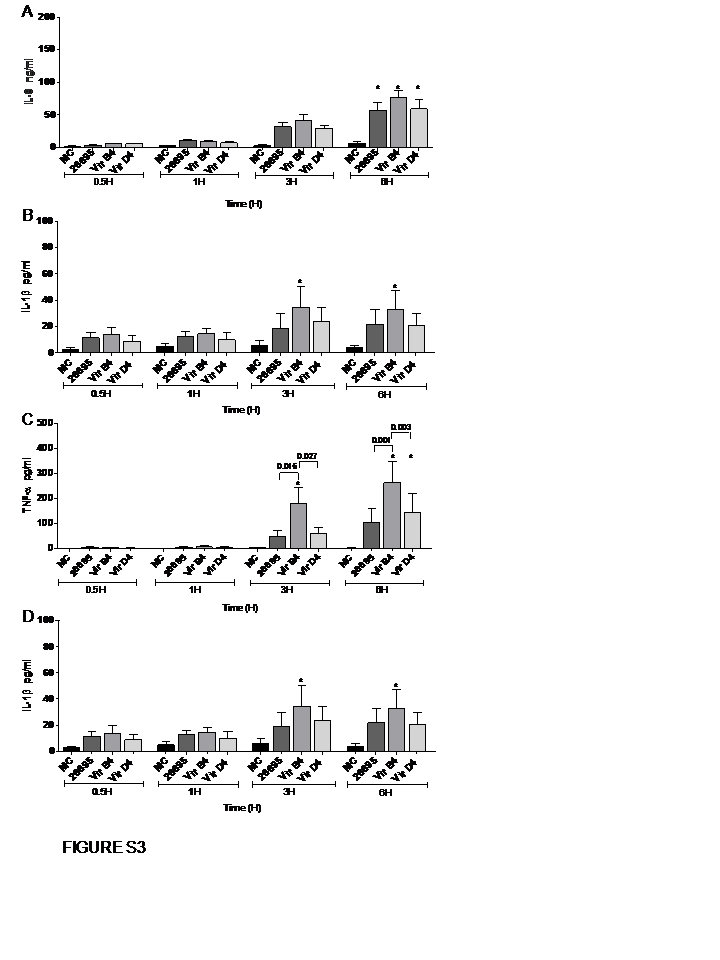

Supplement: Figure S3 — Effect of the integrity of H. pylori T4SS on cytokine production in human neutrophils. A) Strains lacking the cagPAI genes that encode VirB4 or VirD4 protein induced neutrophil IL8 secretion 6 h after infection, which did not differ from the secretion of IL8 induced by the cagPAI+ strain 26695. B) At 3 h and 6 h after infection, the virB4– strain induced a higher level of IL1β secretion than the other mutant virD4– strain or the wild-type cagPAI+ strain. C) At 3 and 6 h, higher levels of TNFα secretion were induced by both mutant strains than by the cagPAI+ strain 26695. D) Both the virB4– and virD4– strains induced similar levels of IL10 secretion, but these did not differ significantly from that induced by cagPAI+ strain 26695. The data shown are from five independent experiments performed in duplicate. The data were assessed with ANOVA to determine their statistical significance. *P<0.05 for H. pylori-infected neutrophils versus uninfected neutrophils. Differences between strains were analyzed with the Student–Newman–Keuls test and were considered statistically significant when *P<0.05. MC = mock control. (TIF) [file pone.0064623.s003.tif]
